# Factors influencing the work of researchers in Scientific Initiation: A systematic review protocol

**DOI:** 10.1371/journal.pone.0297186

**Published:** 2024-01-31

**Authors:** Woska Pires da Costa, Michele da Silva Valadão Fernandes, Aamir Raoof Memon, Priscilla Rayanne E. Silva Noll, Marcos de Moraes Sousa, Matias Noll

**Affiliations:** 1 Instituto Federal Goiano—Campus Ceres, Ceres, Goiás, Brazil; 2 Instituto Federal Goiano—Campus Morrinhos, Morrinhos, Goiás, Brazil; 3 City Hall of São Luís de Montes Belos, Montes Belos, Goiás, Brazil; 4 State Education System—State of Goiás, Brazil; 5 Institute for Health and Sport, Victoria University, Melbourne, Australia; 6 Faculdade de Medicina, Universidade de São Paulo (USP), São Paulo, Brazil; 7 Universidade Federal de Goiás (UFG), Goiânia, Goiás, Brazil; Covenant University, NIGERIA

## Abstract

**Introduction:**

Scientific Initiation (SI) is an educational activity that allows students to begin their scientific training and research under the guidance of an experienced researcher. While several studies have examined students’ perceptions of SI, research on the perspective of researchers working in this field is currently lacking. Thus, this study’s aim is to describe the protocol design for conducting a systematic review. At the same time, the review aims also to identify factors influencing the work of researchers in SI and explore the motivations leading researchers to engage in research projects within institutions and their respective impacts.

**Method and analyses:**

Literature search will be done using the bibliographic databases, including Academic Search Premier, APA PsycNet, CINAHL Plus, ERIC, SocINDEX, Scopus, and Web of Science. The search strategy was guided by the PICo framework (Population, phenomenon of Interest, and Context). The preparation and development of this protocol following guidelines were employed: Preferred Reporting Items for Systematic Reviews and Meta-Analyses for Protocols 2015 (PRISMA-P 2015), Peer Review of Electronic Search Strategies 2015 (PRESS 2015), and PRISMA-Search (PRISMA-S). Original, peer-reviewed articles that examine the factors related to the work of researchers in SI will be included without any language or publication date restrictions. Qualitative, quantitative, and mixed-methods studies will be screened by two independent researchers. The included studies will be analyzed to identify factors, policies, and their impacts obtained analytically. Findings will be objectively categorized and synthetically represented through figures, diagrams, and graphic models. The risk of bias will be assessed using the Critical Appraisal Skills Program (CASP) and the Downs and Black checklists. A third senior reviewer will resolve any discrepancies.

**Discussion:**

We aim to understand the factors that drive researchers to engage in SI research through the dissemination of the findings of this systematic review. This may aid the development of institutional strategies and actions that can support the enhancement of SI programs and encourage greater researcher participation.

## Introduction

Scientific Initiation (SI) is an educational activity that introduces students to the process of scientific knowledge generation [1]. The SI programs, also known as Undergraduate Research (UR) programs at the higher school level, select research projects proposed by advisors and students for execution [2]. It provides them with the fundamental knowledge, methods, techniques, and approaches used in science [3]. In high-income countries such as the United States, Australia, New Zealand, Canada, Japan, and Korea, as well as in several European nations, there is a focus on the promotion of young people’s participation in Science, Technology, Engineering, and Mathematics (STEM) fields through specialized schools and programs that begin in high school [4]. The mentioned countries and others have developed strategic national STEM policy frameworks to remain or become globally competitive [5].

Initiatives in scientific and technological policies in the European Union (EU), starting with the first Framework Programme (FP) in 1984 up to the Horizon 2020 program in 2014 –considered the world’s most extensive research and innovation initiative–demonstrate the commitment of the region’s leaders to drive innovation, address social issues, and promote long-term strategic policies for sustainable research and development [6]. These initiatives are emphasized for their importance in addressing economic challenges, such as low growth and unemployment, following the 2008 financial crisis and for promoting effective long-term strategic policies [7].

In general, there are two ways to generate scientific knowledge: it can be driven by academic interests within a discipline (i.e., the “curiosity-oriented” research), or it can be fueled by societal needs, emerging from a transdisciplinary social and economic context akin to a strategic process [8, 9]. In both cases, funding through public policies is essential [10]. For social, economic, or political purposes, fostering research can influence scientific investigation conducted by research institutions through financial incentives [11–13]. Funding agencies play a vital role in shaping research behavior guiding policies aimed at scientific and technological education to generate social impact and promote international integration [10].

Similarly to the scientific programs mentioned earlier, despite their proportional nature, initiatives are being implemented in low- and middle-income countries, such as Colombia, India, South Africa, and Brazil, to foster SI by providing grants for elementary, high school, and undergraduate students [14]. This was the approach some of these countries adopted to inspire young students from an early age and encourage them to pursue a scientific career. Thus, SI students are encouraged to act as researchers to encourage and prepare them for graduate school and foster new skills for a scientific career [1, 3, 15]. Since scientific knowledge is shaped by the society and culture in which it is produced [16], several countries have implemented programs that provide students with hands-on experience in the scientific field. In this regard, the SI aims to contribute to the training of human resources for research and toward optimizing the time required for completing master’s and doctoral degrees [14, 17]. Furthermore, a study has already indicated that research activities foster the comprehensive development of students in academic, scientific, personal, and professional aspects, ultimately leading to critical and emancipatory actions in society [2].

In the context of the United States, a study examined the individual and institutional factors that predicted the likelihood of STEM field faculty members’ involvement in the research projects of their undergraduate students [18]. According to the study, unfavorable aspects of the research lab environment and infrastructure can potentially lead to students’ decisions to continue scientific research during their studies [19]. These aspects may also impact research supervisors. While it is recognized that mentoring relationships can provide mutual benefits for both research supervisors and students [20], concrete evidence that supports this finding is lacking [18].

Learning opportunities in authentic research environments (e.g., laboratories, experimental areas, university hospitals) should be structured to allow beginner students to understand the routine involved in producing new scientific knowledge [21]. SI students can observe how experiments are designed and developed, how data are analyzed and interpreted, and how theories are tested [21]. However, the supervisor’s experience is a crucial factor in this regard, as a lack of understanding or mastery of the epistemology of science can lead beginner students to adopt a naive perspective of scientific investigation [21]. To enhance student engagement in these programs, higher education institutions must provide adequate resources, quality guidance, and recognition for their work, creating conditions that expand the participation of our students in scientific research across various fields [2].

Therefore, to develop strategies and actions that can improve SI programs, it is essential to understand what drives researchers at educational institutions to engage in scientific research with student participation. The science and technology policies play an essential role in promoting interactions among various agents, enhancing the efficiency of public research institutions, and providing researchers with the autonomy to define their research agendas [22]. In this context, a study conducted in China revealed that scientific and technological innovation policies must establish effective interactions between society and the government to succeed in economic and social development. This synergistic interaction is considered a crucial factor for achieving proposed objectives and effectively driving the scientific and technological progress of the country [23]. Science and technology policies combined are a critical factor in strengthening the national strategic force for the development of modern countries and sustainable economic development [24]. If well-established, these policies would maximize knowledge yield, thus benefiting the wider society. Despite its significance, SI research remains limited, and the evidence to support these activities is lacking.

Given these considerations, it is crucial to emphasize that our study fills a significant gap in the literature. A comprehensive literature search revealed no existing systematic reviews addressing our research objective. This underscores the importance and originality of our study in investigating the positive and negative factors that influence researchers’ work in SI. To provide a clear and comprehensive understanding of these factors, we have formulated the following research guiding question: “What are the factors that influence the work of researchers in Scientific Initiation?”. Subsequently, this manuscript outlines our systematic review protocol, wherein we aim to identify these factors to understand what motivated them to engage in research projects within these institutions and their respective impacts. As part of our focus, we will secondarily extract information on science and technology-related policies from the studies to be included in this systematic review.

## Method

Despite traditionally favoring quantitative evidence in systematic reviews [25], the analysis of qualitative evidence can enhance the findings of quantitative studies by offering a more comprehensive understanding of the impact of contextual factors [26, 27]. Consequently, in recent years, there has been a growing interest in synthesizing evidence derived from studies with diverse designs [25]. This approach can yield a rich, efficient understanding of complex programs [28, 29].

Thus, this systematic review will include quantitative, qualitative, and mixed-methods studies [30], if they meet the eligibility criteria, encompassing associated findings identified by the authors, including those determined through statistical analyses. With this comprehensive approach, we will aspire to identify a broader range of associated factors, which may be described qualitatively, quantitatively, or both, depending on the method approach of the included studies.

### Protocol and registration

This systematic review protocol has been developed according to the guidelines of the Preferred Reporting Items for Systematic Reviews and Meta-Analyses for Protocols 2015 (PRISMA-P 2015) [21, 31, 32]. PRISMA-P 2015 is a checklist of items that aid the preparation and development of a systematic review protocol [31] (S1 File).

After the research question was defined, we initially searched the primary international databases to identify existing studies that focused on SI. This initial search defined the scope of our systematic review and informed us of the development of this protocol, including the methods and analyses that would be used [33].

This protocol has been preregistered on the Open Science Framework platform (OSF Registries, available at: https://doi.org/10.17605/osf.io/upkzw) [34]. Any changes to this protocol during the study will be updated in OSF Registries and reported in the final manuscript that presents the systematic review results [35].

### Search strategy and databases

To ensure a comprehensive global perspective, we selected databases with international coverage, both multidisciplinary and specific scope. For this systematic review, to identify and collect relevant literature, we will search the following seven databases: Academic Search^™^ Premier (ASP), American Psychological Association (APA) PsycNet^®^, Cumulative Index to Nursing and Allied Health Literature^®^ (CINAHL^®^ Plus), Educational Resources Information Center (ERIC), SocINDEX^™^, Scopus^™^, and the Web of Science^™^ Core Collection. The last two listed have a multidisciplinary scope. The search will be conducted in January 2024. Secondary searches will also be performed by reviewing the included studies’ reference lists and relevant systematic reviews. There will be no language or publication date restrictions.

The PICo framework (acronymous of Population, phenomenon of Interest, and Context) [36–38] will be used as the study focuses on Human Sciences field experiences and social phenomena [39], as a guide to formulate the review question [36, 38]. Reviews containing qualitative evidence concentrate on the engagement between the participant and the intervention, which may describe an intervention, but their research question focuses on the perspective of individuals experiencing it as part of a larger phenomenon [36] or context. The PICo strategy was facilitative since the review question requires no intervention or a control group [40]. Moreover, this framework strives to contribute to conducting a descriptive nature review [36], similar to the one we are undertaking.

The guiding keywords block for this study are: P = "researchers" and correlates terms, I = "associated factors" and correlates terms, and Co = "scientific initiation in educational institutions" and correlates terms. The search will be conducted using the following search fields: titles, abstracts, and keywords. To identify all relevant articles, we will use indexed terms and their synonyms and applied Boolean operators and descriptors as described in this systematic review protocol. The Boolean operator "OR" was used to combine similar terms, expanding the reach of the search strategy corresponding to each of the blocks. Subsequently, the blocks were combined using the "AND" operator (Table 1).

**Table 1:** Keywords included in the search strategy are organized in blocks.

Blocks (PICo)	
#1 P	researcher* OR mentor* OR supervisor* OR adviser* OR advisor* OR scientist* OR professor*
#2 I	"associate factor" OR "associates factors" OR "associated factor" OR "associated factors" OR "factor associated" OR "factors associated" OR "related factor" OR "related factors" OR "factor related" OR "factors related" OR "correlated factor" OR "correlated factors" OR "factor correlated" OR "factors correlated" OR "co-related factor" OR "co-related factors" OR "factor co-related" OR "factors co-related" OR "intrinsic factor" OR "intrinsic factors" OR "extrinsic factor" OR "extrinsic factors" OR "individual factor" OR "individual factors" OR "personal factor" OR "personal factors" OR "staff factor" OR "staff factors" OR "motivational factor" OR "motivational factors" OR "motivation factor" OR "motivation factors" OR "motivating factor" OR "motivating factors" OR "motivated factor" OR "motivated factors" OR "motivator factor" OR "motivator factors" OR "external factor" OR "external factors" OR "outer factor" OR "outer factors" OR "internal factor" OR "internal factors" OR "inner factor" OR "inner factors" OR "institutional factor" OR "institutional factors" OR "influence factor" OR "influence factors" OR "factor of influence" OR "factors of influence" OR "induction factor" OR "induction factors" OR "interfering factor" OR "interfering factors" OR "interference factor" OR "interference factors"
#3 Co	((scientific OR science OR research) AND (initiation OR education* OR literacy OR training OR abilit* OR vocation* OR skill* OR instruction* OR background OR internship* OR scholarship* OR studentship* OR "talent development")) OR (scientific AND research)
#4 Co	(education* AND institut*) OR facult* OR universit* OR college* OR "higher education" OR "secondary education" OR "high school" OR "secondary school"
**Search string**:	(#1) AND (#2) AND (#3) AND (#4)

Note: PICo is a strategy to aid in scoping the review, which defines the key elements of the research, i.e., ’P’ to delineate the Problem/Population, ’I’ to specify the phenomenon of Interest, and ’Co’ to detail the Context.

To ensure that the search strategy can effectively identify relevant articles in the databases, we used the evidence-based checklist from the Peer Review of Electronic Search Strategies 2015 (PRESS 2015) guideline, which provides guidance and recommendations for the development of accurate and effective search strategies for electronic literature [41, 42]. Likewise, this guideline’s objectives are to enhance the peer review of electronic literature search strategies, identify search errors, and improve the selection of search terms [42]. It consists of four components: a checklist, a set of six recommendations for librarian practice, a set of four implementation strategies, and an evaluation form.

The validation of the search strategy underwent peer review (by MN, PRESN, and a librarian-documentalist) using the PRESS 2015 checklist. The effectiveness of retrieving relevant studies from electronic databases (i.e., those considered for this systematic review) was verified and validated. In addition, the reviewers (WPC and MN) conducted return tests for each term in the search strategy. The terms that yielded no results were eliminated, and adjustments were overly broad terms. Subsequently, they tested each block individually and, ultimately, the entire search string. These return tests allowed for the identification of the behavior of each database for singular and plural terms, terms composed of more than one word, the use of truncation characters, and other issues related to the specificities of each of the databases considered for this study.

Finally, to ensure that the search strategy was optimized for each database, we applied specific refinement filters to eliminate studies that did not meet the eligibility criteria for this systematic review (S2 File). The guidelines of the PRISMA-Search checklist (PRISMA-S) will be followed during the extraction of metadata from the databases using the search strategy [41]. PRISMA-S covers various aspects of the literature search process for systematic reviews; these include database-specific details, search strategy (registration of limits, restrictions, filters used, etc.), and the documentation process for retrieved and deduplicated records [43].

### Eligibility criteria

This systematic review will include relevant articles with no language restrictions or publication dates that meet the inclusion criteria. Some eligible articles may be excluded if they meet at least one of the previously defined exclusion criteria. Considering the wide variation in the propagation of retractions, which can lead to retracted articles being cited and considered in studies, it is crucial to practice retractions checking [44]. Therefore, the eligible studies that are included in the systematic review will be carefully examined using the Scite–an acronym for “Smart citation index”, available online (https://scite.ai/) [45]–to confirm the validity of the evidence and identify any retraction records [41, 45].

Scite is an online tool that utilizes text mining and artificial intelligence (AI) algorithms to perform article citation counts [46]. It can be employed to contrast citation statements, including contradictory and discrepant claims [45, 46]. Among its various functionalities, Scite verifies whether a particular article has been retracted or disputed [46].

Inclusion criteria:

(i1) Peer-reviewed journal articles published as original studies [47, 48].
(i2) Studies on undergraduate or high school SI level within educational institutions.
(i3) Studies that explore factors influencing the work of researchers in undergraduate or high school SI levels (such as financial rewards, access to funding, working conditions, resources and infrastructure for research development, collaborations, prospects, professional status, vocational or ideological motivation, influential ecosystem for scientific advancement, and compliance with contractual obligations related to work, among others).

Exclusion criteria:

(e1) Duplicates: If multiple articles have been published by the same author, on the same dataset, and on the same topic, only the most comprehensive among them will be considered. Duplicates will be removed following the Bramer’s method [49], and we will conduct a manual review to confirm their exclusion [47, 50].
(e2) Opinion articles, commentaries, editorials or similar, dissertations, theses, reviews, animal studies, articles in press, journal letters, and books [51].
(e3) Studies that are not fully available in the searched databases and that cannot be accessed even after attempts to contact the authors have been made [47, 48].
(e4) Articles written in a restricted language that cannot be appropriately translated [41]. This criterion will only be applied if we exhaust all translation possibilities, such as i) seeking support from our international collaboration network, ii) employing artificial intelligence tools, and iii) hiring specialized companies for the necessary translations. We will indicate this in the findings of our systematic review.
(e5) Studies that have been previously retracted [41].

### Review process

After the completion of the search strategy, the identified articles will be selected, and the metadata will be transferred to the EndNote X9 software (Clarivate, PA, Philadelphia) in RIS format. Duplicate articles will be eliminated using the EndNote software [50, 52]. The metadata will then be imported into the Rayyan^®^ software, available online (https://www.rayyan.ai/), with the reviewers’ blinding feature activated for evaluation [41].

The Rayyan^®^ software stands out for its use of AI, learning from users’ decisions to build a model for predicting studies during screening. This software can remove studies that meet specific criteria, create labels for citations, perform the extraction of relevant words, and use visual and filtering resources to speed up the screening process [53]. Additionally, this software allows reviewers to work in parallel, independently, and with blinding. In summary, Rayyan^®^ is widely used by researchers and allows for greater efficiency in selecting studies for systematic reviews [54]. Considering these and other available features, we have chosen to use Rayyan^®^ software for the screening and selection of studies through the examination of titles and abstracts.

**Fig 1:**
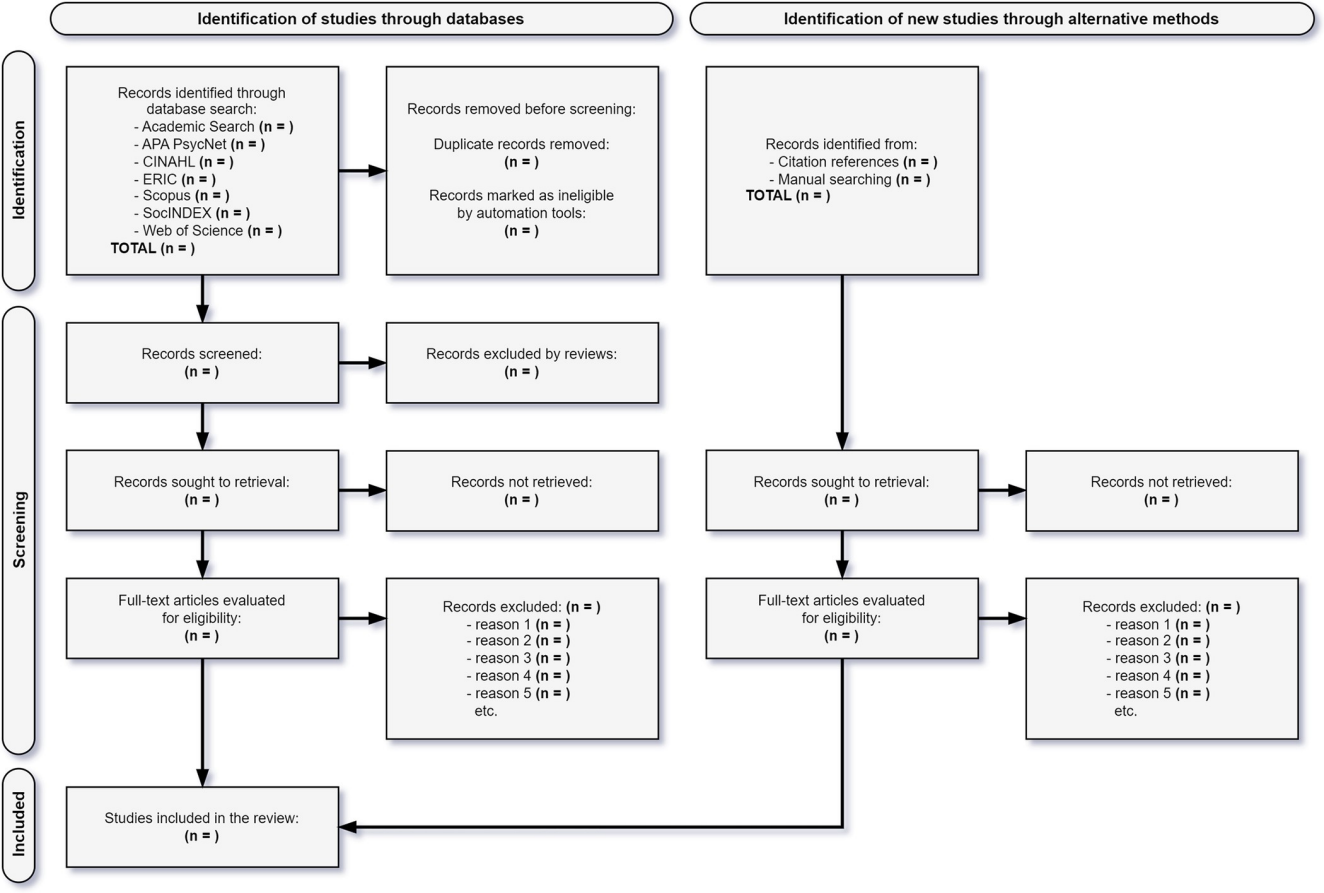
PRISMA 2020 flow diagram for the identification, screening, and inclusion of studies in the review.

Two independent reviewers (WPC and MSVF) will first screen the article based on the titles and abstracts [35, 41, 55]. Any disagreements between the reviewers will be resolved by a senior researcher (MN) [35, 55]. Then, the full texts of the potential articles will be read and reviewed by two independent reviewers (WPC and MSVF), and any discrepancies will be resolved by a third researcher (MN). The exact process will be adopted for assessing the risk of bias in the studies [56]. After completing this process, the selected articles will be included in the systematic review. The flowchart for the selecting studies process on behalf of this systematic review is illustrated in Fig 1 [57].

Conducting a systematic review with independent and parallel reviewers with a blinded process is necessary to eliminate bias in the review process [58]. However, to ensure the transparency and quality of this review, we will assess the inter-rater reliability for the classification of individual components, which will be determined by calculating the percentage of agreement and Cohen’s kappa coefficient [59, 60]. The ratio of agreement and Cohen’s kappa coefficient (denoted by *κ*) will be calculated using the software Statistical Package for the Social Sciences (IBM^™^ SPSS^™^ Statistics), version 26 for Microsoft Windows^™^ (IBM Corp., NY, Armonk) [48]. The Cohen’s kappa coefficient equation is presented as Eq (1) [61]:

$$\kappa =\frac{P_{o}-P_{e}}{1-P_{e}}$$ (1)

In this equation, the variable *P*_*o*_ is the observed proportion of agreement among the reviewers, and *P*_*e*_ is the expected proportion of chance agreement. The *κ* value is theoretically between −1 and 1. If the reviewers make the same decision for all items, the *κ* value would be 1 [60]. Common interpretations are as follows [61]:

- *κ* = 1→indicates perfect agreement.
- *κ*>0→indicates agreement beyond what would be expected by chance.
- *κ* = 0→indicates agreement equals chance agreement.
- *κ*<0→indicates disagreement beyond what would be expected by chance.

### Data extraction, synthesis, and analysis

Two independent reviewers (WPC and MSVF) will extract, assess the data, synthesize the qualitative and quantitative data, and any discrepancies will be resolved by a third reviewer (MN). Both primary reviewers will carry out this entire process simultaneously and in a blinded manner. If necessary, one of the researchers (WPC) will attempt to contact the authors to obtain any missing relevant data related to the included studies [56].

To extract data for this study, a spreadsheet that includes the aspects detailed in Table 2 will be used to summarize and explain the characteristics and findings of the included studies [62]. Information will be extracted from the included studies, such as publication details (e.g., title, year, journal, DOI), context in which the study was conducted (e.g., country, institution, education level, knowledge area, date, scope, population involved in the study), methodological design (e.g., type of analysis, sample size), and obtained results (e.g., factors associated with researchers’ work, policies related to science and technology, and their respective impacts).

**Table 2:** Primary version of the data extraction table of the studies included in this systematic review.

#	Surname of the first author	Date of Publication	Study title / DOI	Journal of Publication	Source of the data (Primary/ Secondary)	Study location	Public addressed	Teaching level	Sample size	Time frame or study period	Instrument used for data collection	Study design (qualitative/ quantitative/ mixed)	Type of analysis performed	Associated factors	Science and technology policies or programs	Impacts	Gaps found
**1**																	
**2**																	
**…**																	
**Hypothetical example**	Mason	2021	Factors influencing researchers from scientific research: a case study 10.1186/a123-00-x	Education Journal	Primary	Germany	Researchers engaged in SI	Undergraduate degree	253	2018–2019	Questionnaire and Interview	Qualitative	Chi-square statistic	a) Institutional factors: - scarce budgetary resources; - limited number of scholarships; - overload of teaching activities. b) Personal factors: - prefers to teach. - does not prioritize research.	German Academic Exchange Service (*Deutscher Akademischer Austauschdienst—DAAD*), or also known as *DAAD* Scholarships Program.	- Promotes the internationalization of research and the training of future scientists with global experiences; - Develops essential skills for participants; - Drives innovative research; - Contributes to the global engagement of German students.	- Training for group work; - Increase investments; - Offer a scientific pre-training course to students who enter the institution.
**…**																	
***n***																	

Note: DOI is an acronym for Digital Object Identifier.

We will employ a mixed-methods approach [29, 63] to synthesize results from primary studies that combine qualitative and quantitative methods [64]. In the case of quantitative and mixed-methods studies, we will encompass associated findings identified by the authors, including those determined through statistical analyses. For quantitative and mixed studies, the quantitative synthesis will be conducted first to quantify the effect of each component, followed by qualitative synthesis to identify relevant features (e.g., the factors, policies, and respective impacts) of interest in our investigation. These components, which have been analytically obtained, will be carried out.

Subsequently, the findings will be categorized (and subcategorized if necessary) in an objective and synthetic manner. Thus, quantitative data will be transformed into categories/themes [25]. These obtained data may also be presented through figures, diagrams, or other graphical elements that best depict the clustering of identified factors, patterns, and trends, as conducted by [2] and/or proposed by [30]. For instance, the visual mapping technique is wealthy and commonly employed to conceptualize process data [65].

Lastly, because of this systematic review, we will also consider conducting a separate quantitative synthesis [37, 66], through meta-analysis in addition to the qualitative synthesis described earlier. Meta-analysis is the statistical synthesis method used to aggregate the results of the reviews [25, 67]. Therefore, the meta-analysis will only be conducted in this study if our investigation’s quantitative findings are sufficient for this analysis.

### Assessment of risk of bias

The Critical Appraisal Skills Program (CASP) is a widely used critical appraisal tool to review and evaluate different types of research studies [68]. This tool has adapted its guidelines to fit each type of study, considering specific characteristics and corresponding methodological approaches, including qualitative studies [68]. The CASP checklist will assess the risk of bias in qualitative studies [69–72]. This tool considers three broad questions in the evaluation: “Are the study results valid?” (Section A), “What are the results?” (Section B), and “Will the results be helpful locally?” (Section C) [68, 70]. The CASP checklist comprises 10 questions [68, 72]–encompassing these three sections–, with nine of them requiring objective marking rated as “yes” (2 points), “can’t tell” (1 point), or “no” (0 points), resulting in a maximum score of 18 [73]. The 10^th^ question requires a qualitative response [70, 73]. The overall quality of each evaluated study will be assigned a score, based on a three-star system, in three categories: low (one star; 0–6 points), moderate (two stars; 7–12 points), and high (three stars; 13–18 points) [73]. A summary table will be generated with the CASP ratings [68].

Quantitative studies will be evaluated using the 27-item Downs and Black checklist [74]. However, considering that some items on this checklist may not apply to observational studies, we will use a different score for cross-sectional (0–12 points) and longitudinal (0–16 points) studies [56, 69]. We have selected a reduced version of the checklist, consisting of 16 items (items: 1–3, 5–7, 9–12, 17, 18, 20, 21, 25, and 26) [69], which has been adapted from the original. A summary quality score for each selected study will be calculated by expressing the score as a percentage of the maximum possible score for the study design [56]. A score of 70% or higher will be considered as “a low risk of bias”, while a score lower than 70% will be regarded as “a high risk of bias” [69, 74].

### Reviewer training

Authors responsible for evaluating article eligibility will be trained in using the inclusion and exclusion criteria, and an eligibility test will be conducted on 50 titles and abstracts before the articles are coded [35, 75, 76]. Rayyan^®^ and EndNote software will be used in the selection process [35, 50]. Rayyan^®^ is a tool that helps with systematic review and meta-analysis research [53], while EndNote is a bibliographic reference manager that can be used for article screening [50, 52]. Additionally, researchers will participate in a training session to standardize the assessment of the risk of bias and learn how to extract data from articles; they will evaluate five studies that will not be included in the final review [35, 76]. This training will also cover the use of EndNote and Rayyan^®^ software for standardized analyses [35].

## Discussion

Scientific knowledge should not be perceived simply as an accumulation of facts but as something socially constructed by groups of scientists through their daily activities and communication with peers [77, 78]. The United Nations (UN) considers scientific research a key change agent in addressing societal challenges [79]. As such, science is important because highly trained human resources are regarded as essential capital that is fundamental for scientific and technological progress in promoting the well-being of a country [80]. For science to be considered a social good, effective scientific communication and dissemination policies must be established [81]. It is no coincidence that many developed nations think modern science, along with its technology and economic system, is a marker of power [82]. This perspective has contributed to dividing the world into nations that produce new knowledge and those that consume it. In this context, the participation of young people in STEM fields is a matter of international concern, and careers that require knowledge of the physical sciences and advanced mathematics are particularly affected by this issue [4].

SI is a training strategy designed to prepare young people for adopting and managing new technologies, as well as pursuing academic and/or scientific careers relevant to training new researchers and scientists, considering the growing global demand for highly qualified professionals [83]. It is recommended that science education be implemented as a state policy to aid certain countries in improving their science, technology, and innovation indicators. Furthermore, it is an activity of potential strategic importance, as the technological growth of a nation is closely tied to the extent to which young students are encouraged to engage in research. The seamless integration of research and teaching is crucial in creating an environment conducive to research [3].

Notably, the encouragement of scientific research should begin in early childhood education and be implemented on a continuous basis [84], either through its integration into the school curricula or through extracurricular projects [83]. Given this recommendation, SI should be treated as a priority training proposal, and the number of related vacancies and opportunities should be expanded as far as possible. Therefore, increasing the number of SI scholarship recipients is desirable to meet the needs of research professors, students, and those engaged in academically meritorious projects. Additionally, institutions may consider it essential to demystify and reduce SI programs’ elitist and exclusionary nature in their countries.

Students must engage in research activities, such as those involving SI, that are important for students to move beyond common sense. When introduced in a school or academic setting, SI enhances the learning process through problem-solving skills [83, 85]. It also helps citizens become scientifically literate and aware of the importance of science and its related public issues. It encourages them to be involved in decision-making on issues such as public health, the use of natural resources, food production and consumption, and environmental protection, among others [86, 87].

### Strengths and limitations

Systematic review studies may have certain limitations. In our investigation, if no studies meet the defined eligibility criteria, the review may be reported as an “empty review” [88]. However, even in this case, the result remains relevant and may stimulate more appropriate future investigations about SI programs [88]. Additionally, the specificity and uniqueness of public policies about SI in each country may make it difficult to assess all studies conducted and those that address the review’s objectives. Additionally, the articles included may have gaps in their methodological procedures, such as unclear or non-validated instruments to identify factors associated with researchers’ work in undergraduate research; this suggests that the search strategy may not capture potentially essential studies for the review. Consequently, the analysis of the results may be difficult, or even impossible, in some cases, not allowing us to grasp the reality of undergraduate research worldwide, particularly concerning the factors.

However, this study has some strengths that should be acknowledged. First, this review uses more complex techniques and a wider range of terms in the search strategy. Of note, the validation of the search strategy is not a commonly considered process in systematic reviews despite it being a recommended practice. Second, the inclusion of seven databases is a positive aspect, as its comprehensiveness would be essential in covering studies from different contexts. Third, there are no language or time constraints in this study. Fourth, the risk of bias will be conducted using established methods. Fifth, the analysis follows a rigorous data extraction and evidence evaluation method, which will be conducted by two trained reviewers independently. Finally, as there have been no prior systematic reviews on this topic, it is expected that this study will contribute to a better understanding of the research question and fill a significant gap in the field of scientometrics.

## Conclusion

In conclusion, we emphasize that the findings of this study can act as a stimulus for further research on this topic and aid the identification of any research gaps. Furthermore, an insight into the factors that affect researchers’ work as advisors can help improve public policies related to SI programs and, in particular, educational management by providing more focused strategies to promote the development of SI research projects. Consequently, the benefits of SI can be more effectively extended by the active involvement of students, researchers, and institutions.

## Supporting information

S1 File The PRISMA-P checklist for this systematic review protocol. (PDF)

S2 File Details of the Boolean search string for each database. (PDF)
