# Supplementary material for: Factors influencing the work of researchers in Scientific Initiation: A systematic review protocol
Source: PLoS One. 2024 Jan 31;19(1):e0297186. doi: 10.1371/journal.pone.0297186 (PMC10829991; doi:10.1371/journal.pone.0297186)
Supplement: S2 File — (PDF) [file pone.0297186.s002.pdf]

S2 File. Details of the Boolean search string for each database.

Web of Science™ (WoS) Core Collection via native interface	
**Blocks and Returns**	**Search strings**
#1<br>(Return: ‹n_1›)	TS=(researcher* OR mentor* OR supervisor* OR adviser* OR advisor* OR scientist* OR professor*)
#2<br>(Return: ‹n_2›)	TS=("associate factor" OR "associates factors" OR "associated factor" OR "associated factors" OR "factor associated" OR "factors associated" OR "related factor" OR "related factors" OR "factor related" OR "factors related" OR "correlated factor" OR "correlated factors" OR "factor correlated" OR "factors correlated" OR "co-related factor" OR "co-related factors" OR "factor co-related" OR "factors co-related" OR "intrinsic factor" OR "intrinsic factors" OR "extrinsic factor" OR "extrinsic factors" OR "individual factor" OR "individual factors" OR "personal factor" OR "personal factors" OR "staff factor" OR "staff factors" OR "motivational factor" OR "motivational factors" OR "motivation factor" OR "motivation factors" OR "motivating factor" OR "motivating factors" OR "motivated factor" OR "motivated factors" OR "motivator factor" OR "motivator factors" OR "external factor" OR "external factors" OR "outer factor" OR "outer factors" OR "internal factor" OR "internal factors" OR "inner factor" OR "inner factors" OR "institutional factor" OR "institutional factors" OR "influence factor" OR "influence factors" OR "factor of influence" OR "factors of influence" OR "induction factor" OR "induction factors" OR "interfering factor" OR "interfering factors" OR "interference factor" OR "interference factors")
#3<br>(Return: ‹n_3›)	TS=(((scientific OR science OR research) AND (initiation OR education* OR literacy OR training OR abilit* OR vocation* OR skill* OR instruction* OR background OR internship* OR scholarship* OR studentship* OR "talent development")) OR (scientific AND research))
#4<br>(Return: ‹n_4›)	TS=((education* AND institut*) OR facult* OR universit* OR college* OR "higher education" OR "secondary education" OR "high school" OR "secondary school")
#5<br>(Return: ‹n_5›)	(#1) AND (#2) AND (#3) AND (#4)
	**Search refinement**:<br>1. Document type: Article<br>**Return after refinement**:<br>‹*N*› studies in the test carried out in ‹*date*›.

Scopus™ via native interface	
**Blocks and Returns**	**Search strings**
#1 (Return: ‹n_1›)	TITLE-ABS-KEY(researcher* OR mentor* OR supervisor* OR adviser* OR advisor* OR scientist* OR professor*)
#2 (Return: ‹n_2›)	TITLE-ABS-KEY("associate factor" OR "associates factors" OR "associated factor" OR "associated factors" OR "factor associated" OR "factors associated" OR "related factor" OR "related factors" OR "factor related" OR "factors related" OR "correlated factor" OR "correlated factors" OR "factor correlated" OR "factors correlated" OR "co-related factor" OR "co-related factors" OR "factor co-related" OR "factors co-related" OR "intrinsic factor" OR "intrinsic factors" OR "extrinsic factor" OR "extrinsic factors" OR "individual factor" OR "individual factors" OR "personal factor" OR "personal factors" OR "staff factor" OR "staff factors" OR "motivational factor" OR "motivational factors" OR "motivation factor" OR "motivation factors" OR "motivating factor" OR "motivating factors" OR "motivated factor" OR "motivated factors" OR "motivator factor" OR "motivator factors" OR "external factor" OR "external factors" OR "outer factor" OR "outer factors" OR "internal factor" OR "internal factors" OR "inner factor" OR "inner factors" OR "institutional factor" OR "institutional factors" OR "influence factor" OR "influence factors" OR "factor of influence" OR "factors of influence" OR "induction factor" OR "induction factors" OR "interfering factor" OR "interfering factors" OR "interference factor" OR "interference factors")
#3 (Return: ‹n_3›)	TITLE-ABS-KEY(((scientific OR science OR research) AND (initiation OR education* OR literacy OR training OR abilit* OR vocation* OR skill* OR instruction* OR background OR internship* OR scholarship* OR studentship* OR "talent development")) OR (scientific AND research))
#4 (Return: ‹n_4›)	TITLE-ABS-KEY((education* AND institut*) OR facult* OR universit* OR college* OR "higher education" OR "secondary education" OR "high school" OR "secondary school")
#5 (Return: ‹n_5›)	(#1) AND (#2) AND (#3) AND (#4)
	**Search refinement**:<br>1. Source type: Journal<br>2. Document type: Article<br>3. Publication stage: Final<br>**Return after refinement**:<br>‹*N*› studies in the test carried out in ‹*date*›.

ERIC via native interface	
**Blocks and Returns**	**Search strings**
#1<br>(Return: ‹n_1›)	title:(researcher OR mentor OR supervisor OR adviser OR advisor OR scientist OR professor) **OR** abstract:(researcher OR mentor OR supervisor OR adviser OR advisor OR scientist OR professor) **OR** descriptor:(researcher OR mentor OR supervisor OR adviser OR advisor OR scientist OR professor)
#2<br>(Return: ‹n_2›)	title:("associate factor" OR "associated factor" OR "factor associated" OR "related factor" OR "factor related" OR "correlated factor" OR "factor correlated" OR "intrinsic factor" OR "extrinsic factor" OR "individual factor" OR "personal factor" OR "staff factor" OR "motivational factor" OR "motivation factor" OR "motivating factor" OR "motivated factor" OR "motivator factor" OR "external factor" OR "outer factor" OR "internal factor" OR "inner factor" OR "institutional factor" OR "influence factor" OR "factor of influence" OR "induction factor" OR "interfering factor" OR "interference factor") **OR** abstract:("associate factor" OR "associated factor" OR "factor associated" OR "related factor" OR "factor related" OR "correlated factor" OR "factor correlated" OR "intrinsic factor" OR "extrinsic factor" OR "individual factor" OR "personal factor" OR "staff factor" OR "motivational factor" OR "motivation factor" OR "motivating factor" OR "motivated factor" OR "motivator factor" OR "external factor" OR "outer factor" OR "internal factor" OR "inner factor" OR "institutional factor" OR "influence factor" OR "factor of influence" OR "induction factor" OR "interfering factor" OR "interference factor") **OR** descriptor:("associate factor" OR "associated factor" OR "factor associated" OR "related factor" OR "factor related" OR "correlated factor" OR "factor correlated" OR "intrinsic factor" OR "extrinsic factor" OR "individual factor" OR "personal factor" OR "staff factor" OR "motivational factor" OR "motivation factor" OR "motivating factor" OR "motivated factor" OR "motivator factor" OR "external factor" OR "outer factor" OR "internal factor" OR "inner factor" OR "institutional factor" OR "influence factor" OR "factor of influence" OR "induction factor" OR "interfering factor" OR "interference factor")
#3<br>(Return: ‹n_3›)	title:(((scientific OR science OR research) AND (initiation OR education OR literacy OR training OR ability OR vocation OR skill OR instruction OR background OR internship OR scholarship OR studentship OR "talent development")) OR (scientific AND research)) **OR** abstract:(((scientific OR science OR research) AND (initiation OR education OR literacy OR training OR ability OR vocation OR skill OR instruction OR background OR internship OR scholarship OR studentship OR "talent development")) OR (scientific AND research)) **OR** descriptor:(((scientific OR science OR research) AND (initiation OR education OR literacy OR training OR ability OR vocation OR skill OR instruction OR background OR internship OR scholarship OR studentship OR "talent development")) OR (scientific AND research))
#4<br>(Return: ‹n_4›)	title:((education AND institute) OR faculty OR university OR college OR "higher education" OR "secondary education" OR "high school" OR "secondary school") **OR** abstract:((education AND institute) OR faculty OR university OR college OR "higher education" OR "secondary education" OR "high school" OR "secondary school") **OR** descriptor:((education AND institute) OR faculty OR university OR college OR "higher education" OR "secondary education" OR "high school" OR "secondary school")
#5<br>(Return: ‹n_5›)	(#1) AND (#2) AND (#3) AND (#4)<br><br>This database does not allow combination of search blocks, which means that the return to block #5 was only possible for a search string that includes all other blocks (i.e., blocks #1, #2, #3 and #4). Another important detail to be reported refers to the character limit that the database accepts as a search string, which, in this particular case, was exceeded. As an alternative to circumvent this limitation, block #5 did not include only the title, abstract and descriptor fields, but all fields, which includes the full text. Therefore, it is expected that, even after applying the refinement of the search, this total obtained may contain a greater number of studies that do not meet the requirements of this research. Below is the string used for block #5:<br><br>(researcher OR mentor OR supervisor OR adviser OR advisor OR scientist OR professor) **AND** ("associate factor" OR "associated factor" OR "factor associated" OR "related factor" OR "factor related" OR "correlated factor" OR "factor correlated" OR "intrinsic factor" OR "extrinsic factor" OR "individual factor" OR "personal factor" OR "staff factor" OR "motivational factor" OR "motivation factor" OR "motivating factor" OR "motivated factor" OR "motivator factor" OR "external factor" OR "outer factor" OR "internal factor" OR "inner factor" OR "institutional factor" OR "influence factor" OR "factor of influence" OR "induction factor" OR "interfering factor" OR "interference factor") **AND** (((scientific OR science OR research) AND (initiation OR education OR literacy OR training OR ability OR vocation OR skill OR instruction OR background OR internship OR scholarship OR studentship OR "talent development")) OR (scientific AND research)) **AND** ((education AND institute) OR faculty OR university OR college OR "higher education" OR "secondary education" OR "high school" OR "secondary school")
	**Search refinement**:<br>1. Peer reviewed only: Yes<br>**Return after refinement**:<br>‹N› studies in the test carried out in ‹*date*›.

SocINDEX™ with Full Text via EBSCOhost™ interface

Blocks and Returns	Search strings
#1 (Return: $\langle n_1 \rangle$)	TI(researcher* OR mentor* OR supervisor* OR adviser* OR advisor* OR scientist* OR professor*) **OR** AB(researcher* OR mentor* OR supervisor* OR adviser* OR advisor* OR scientist* OR professor*) **OR** KW(researcher* OR mentor* OR supervisor* OR adviser* OR advisor* OR scientist* OR professor*)
#2 (Return: $\langle n_2 \rangle$)	TI("associate factor" OR "associates factors" OR "associated factor" OR "associated factors" OR "factor associated" OR "factors associated" OR "related factor" OR "related factors" OR "factor related" OR "factors related" OR "correlated factor" OR "correlated factors" OR "factor correlated" OR "factors correlated" OR "co-related factor" OR "co-related factors" OR "factor co-related" OR "factors co-related" OR "intrinsic factor" OR "intrinsic factors" OR "extrinsic factor" OR "extrinsic factors" OR "individual factor" OR "individual factors" OR "personal factor" OR "personal factors" OR "staff factor" OR "staff factors" OR "motivational factor" OR "motivational factors" OR "motivation factor" OR "motivation factors" OR "motivating factor" OR "motivating factors" OR "motivated factor" OR "motivated factors" OR "motivator factor" OR "motivator factors" OR "external factor" OR "external factors" OR "outer factor" OR "outer factors" OR "internal factor" OR "internal factors" OR "inner factor" OR "inner factors" OR "institutional factor" OR "institutional factors" OR "influence factor" OR "influence factors" OR "factor of influence" OR "factors of influence" OR "induction factor" OR "induction factors" OR "interfering factor" OR "interfering factors" OR "interference factor" OR "interference factors") **OR** AB("associate factor" OR "associates factors" OR "associated factor" OR "associated factors" OR "factor associated" OR "factors associated" OR "related factor" OR "related factors" OR "factor related" OR "factors related" OR "correlated factor" OR "correlated factors" OR "factor correlated" OR "factors correlated" OR "co-related factor" OR "co-related factors" OR "factor co-related" OR "factors co-related" OR "intrinsic factor" OR "intrinsic factors" OR "extrinsic factor" OR "extrinsic factors" OR "individual factor" OR "individual factors" OR "personal factor" OR "personal factors" OR "staff factor" OR "staff factors" OR "motivational factor" OR "motivational factors" OR "motivation factor" OR "motivation factors" OR "motivating factor" OR "motivating factors" OR "motivated factor" OR "motivated factors" OR "motivator factor" OR "motivator factors" OR "external factor" OR "external factors" OR "outer factor" OR "outer factors" OR "internal factor" OR "internal factors" OR "inner factor" OR "inner factors" OR "institutional factor" OR "institutional factors" OR "influence factor" OR "influence factors" OR "factor of influence" OR "factors of influence" OR "induction factor" OR "induction factors" OR "interfering factor" OR "interfering factors" OR "interference factor" OR "interference factors") **OR** KW("associate factor" OR "associates factors" OR "associated factor" OR "associated factors" OR "factor associated" OR "factors associated" OR "related factor" OR "related factors" OR "factor related" OR "factors related" OR "correlated factor" OR "correlated factors" OR "factor correlated" OR "factors correlated" OR "co-related factor" OR "co-related factors" OR "factor co-related" OR "factors co-related" OR "intrinsic factor" OR "intrinsic factors" OR "extrinsic factor" OR "extrinsic factors" OR "individual factor" OR "individual factors" OR "personal factor" OR "personal factors" OR "staff factor" OR "staff factors" OR "motivational factor" OR "motivational factors" OR "motivation factor" OR "motivation factors" OR "motivating factor" OR "motivating factors" OR "motivated factor" OR "motivated factors" OR "motivator factor" OR "motivator factors" OR "external factor" OR "external factors" OR "outer factor" OR "outer factors" OR "internal factor" OR "internal factors" OR "inner factor" OR "inner factors" OR "institutional factor" OR "institutional factors" OR "influence factor" OR "influence factors" OR "factor of influence" OR "factors of influence" OR "induction factor" OR "induction factors" OR "interfering factor" OR "interfering factors" OR "interference factor" OR "interference factors")
#3 (Return: $\langle n_3 \rangle$)	TI(((scientific OR science OR research) AND (initiation OR education* OR literacy OR training OR abilit* OR vocation* OR skill* OR instruction* OR background OR internship* OR scholarship* OR studentship* OR "talent development")) OR (scientific AND research)) **OR** AB(((scientific OR science OR research) AND (initiation OR education* OR literacy OR training OR abilit* OR vocation* OR skill* OR instruction* OR background OR internship* OR scholarship* OR studentship* OR "talent development")) OR (scientific AND research)) **OR** KW(((scientific OR science OR research) AND (initiation OR education* OR literacy OR training OR abilit* OR vocation* OR skill* OR instruction* OR background OR internship* OR scholarship* OR studentship* OR "talent development")) OR (scientific AND research))
#4 (Return: $\langle n_4 \rangle$)	TI((education* AND institut*) OR facult* OR universit* OR college* OR "higher education" OR "secondary education" OR "high school" OR "secondary school") **OR** AB((education* AND institut*) OR facult* OR universit* OR college* OR "higher education" OR "secondary education" OR "high school" OR "secondary school") **OR** KW((education* AND institut*) OR facult* OR universit* OR college* OR "higher education" OR "secondary education" OR "high school" OR "secondary school")
#5 (Return: $\langle n_5 \rangle$)	(#1) AND (#2) AND (#3) AND (#4)

Search refinement:

1. Search Expanders: Apply related words and Apply equivalent subjects
2. Limit for: Academic journals (reviewed by experts)
3. Types of documents: Academic journals

Return after refinement

$\langle N \rangle$ studies in the test carried out in $\langle date \rangle$.

Academic Search™ Premier (ASP) via EBSCOhost™ interface

Blocks and Returns	Search strings
#1<br>(Return: ‹n_1›)	TI(researcher* OR mentor* OR supervisor* OR adviser* OR advisor* OR scientist* OR professor*) **OR** AB(researcher* OR mentor* OR supervisor* OR adviser* OR advisor* OR scientist* OR professor*) **OR** KW(researcher* OR mentor* OR supervisor* OR adviser* OR advisor* OR scientist* OR professor*)
#2<br>(Return: ‹n_2›)	TI("associate factor" OR "associates factors" OR "associated factor" OR "associated factors" OR "factor associated" OR "factors associated" OR "related factor" OR "related factors" OR "factor related" OR "factors related" OR "correlated factor" OR "correlated factors" OR "factor correlated" OR "factors correlated" OR "co-related factor" OR "co-related factors" OR "factor co-related" OR "factors co-related" OR "intrinsic factor" OR "intrinsic factors" OR "extrinsic factor" OR "extrinsic factors" OR "individual factor" OR "individual factors" OR "personal factor" OR "personal factors" OR "staff factor" OR "staff factors" OR "motivational factor" OR "motivational factors" OR "motivation factor" OR "motivation factors" OR "motivating factor" OR "motivating factors" OR "motivated factor" OR "motivated factors" OR "motivator factor" OR "motivator factors" OR "external factor" OR "external factors" OR "outer factor" OR "outer factors" OR "internal factor" OR "internal factors" OR "inner factor" OR "inner factors" OR "institutional factor" OR "institutional factors" OR "influence factor" OR "influence factors" OR "factor of influence" OR "factors of influence" OR "induction factor" OR "induction factors" OR "interfering factor" OR "interfering factors" OR "interference factor" OR "interference factors") **OR** AB("associate factor" OR "associates factors" OR "associated factor" OR "associated factors" OR "factor associated" OR "factors associated" OR "related factor" OR "related factors" OR "factor related" OR "factors related" OR "correlated factor" OR "correlated factors" OR "factor correlated" OR "factors correlated" OR "co-related factor" OR "co-related factors" OR "factor co-related" OR "factors co-related" OR "intrinsic factor" OR "intrinsic factors" OR "extrinsic factor" OR "extrinsic factors" OR "individual factor" OR "individual factors" OR "personal factor" OR "personal factors" OR "staff factor" OR "staff factors" OR "motivational factor" OR "motivational factors" OR "motivation factor" OR "motivation factors" OR "motivating factor" OR "motivating factors" OR "motivated factor" OR "motivated factors" OR "motivator factor" OR "motivator factors" OR "external factor" OR "external factors" OR "outer factor" OR "outer factors" OR "internal factor" OR "internal factors" OR "inner factor" OR "inner factors" OR "institutional factor" OR "institutional factors" OR "influence factor" OR "influence factors" OR "factor of influence" OR "factors of influence" OR "induction factor" OR "induction factors" OR "interfering factor" OR "interfering factors" OR "interference factor" OR "interference factors") **OR** KW("associate factor" OR "associates factors" OR "associated factor" OR "associated factors" OR "factor associated" OR "factors associated" OR "related factor" OR "related factors" OR "factor related" OR "factors related" OR "correlated factor" OR "correlated factors" OR "factor correlated" OR "factors correlated" OR "co-related factor" OR "co-related factors" OR "factor co-related" OR "factors co-related" OR "intrinsic factor" OR "intrinsic factors" OR "extrinsic factor" OR "extrinsic factors" OR "individual factor" OR "individual factors" OR "personal factor" OR "personal factors" OR "staff factor" OR "staff factors" OR "motivational factor" OR "motivational factors" OR "motivation factor" OR "motivation factors" OR "motivating factor" OR "motivating factors" OR "motivated factor" OR "motivated factors" OR "motivator factor" OR "motivator factors" OR "external factor" OR "external factors" OR "outer factor" OR "outer factors" OR "internal factor" OR "internal factors" OR "inner factor" OR "inner factors" OR "institutional factor" OR "institutional factors" OR "influence factor" OR "influence factors" OR "factor of influence" OR "factors of influence" OR "induction factor" OR "induction factors" OR "interfering factor" OR "interfering factors" OR "interference factor" OR "interference factors")
#3<br>(Return: ‹n_3›)	TI(((scientific OR science OR research) AND (initiation OR education* OR literacy OR training OR abilit* OR vocation* OR skill* OR instruction* OR background OR internship* OR scholarship* OR studentship* OR "talent development")) OR (scientific AND research)) **OR** AB(((scientific OR science OR research) AND (initiation OR education* OR literacy OR training OR abilit* OR vocation* OR skill* OR instruction* OR background OR internship* OR scholarship* OR studentship* OR "talent development")) OR (scientific AND research)) **OR** KW(((scientific OR science OR research) AND (initiation OR education* OR literacy OR training OR abilit* OR vocation* OR skill* OR instruction* OR background OR internship* OR scholarship* OR studentship* OR "talent development")) OR (scientific AND research))
#4<br>(Return: ‹n_4›)	TI((education* AND institut*) OR facult* OR universit* OR college* OR "higher education" OR "secondary education" OR "high school" OR "secondary school") **OR** AB((education* AND institut*) OR facult* OR universit* OR college* OR "higher education" OR "secondary education" OR "high school" OR "secondary school") **OR** KW((education* AND institut*) OR facult* OR universit* OR college* OR "higher education" OR "secondary education" OR "high school" OR "secondary school")
#5<br>(Return: ‹n_5›)	(#1) AND (#2) AND (#3) AND (#4)

Search refinement:

1. Search Expanders: Apply related words and Apply equivalent subjects
2. Limit for: Academic journals (reviewed by experts)
3. Types of documents: Academic journals

Return after refinement:

‹*N*› studies in the test carried out in ‹*date*›.

CINAHL® Plus with Full Text via EBSCOhost™ interface

**Blocks and Returns**	**Search strings**
#1<br>(Return: ‹*n_1*›)	TI(researcher* OR mentor* OR supervisor* OR adviser* OR advisor* OR scientist* OR professor*) **OR** AB(researcher* OR mentor* OR supervisor* OR adviser* OR advisor* OR scientist* OR professor*) **OR** MW(researcher* OR mentor* OR supervisor* OR adviser* OR advisor* OR scientist* OR professor*)
#2<br>(Return: ‹*n_2*›)	TI("associate factor" OR "associates factors" OR "associated factor" OR "associated factors" OR "factor associated" OR "factors associated" OR "related factor" OR "related factors" OR "factor related" OR "factors related" OR "correlated factor" OR "correlated factors" OR "factor correlated" OR "factors correlated" OR "co-related factor" OR "co-related factors" OR "factor co-related" OR "factors co-related" OR "intrinsic factor" OR "intrinsic factors" OR "extrinsic factor" OR "extrinsic factors" OR "individual factor" OR "individual factors" OR "personal factor" OR "personal factors" OR "staff factor" OR "staff factors" OR "motivational factor" OR "motivational factors" OR "motivation factor" OR "motivation factors" OR "motivating factor" OR "motivating factors" OR "motivated factor" OR "motivated factors" OR "motivator factor" OR "motivator factors" OR "external factor" OR "external factors" OR "outer factor" OR "outer factors" OR "internal factor" OR "internal factors" OR "inner factor" OR "inner factors" OR "institutional factor" OR "institutional factors" OR "influence factor" OR "influence factors" OR "factor of influence" OR "factors of influence" OR "induction factor" OR "induction factors" OR "interfering factor" OR "interfering factors" OR "interference factor" OR "interference factors") **OR** AB("associate factor" OR "associates factors" OR "associated factor" OR "associated factors" OR "factor associated" OR "factors associated" OR "related factor" OR "related factors" OR "factor related" OR "factors related" OR "correlated factor" OR "correlated factors" OR "factor correlated" OR "factors correlated" OR "co-related factor" OR "co-related factors" OR "factor co-related" OR "factors co-related" OR "intrinsic factor" OR "intrinsic factors" OR "extrinsic factor" OR "extrinsic factors" OR "individual factor" OR "individual factors" OR "personal factor" OR "personal factors" OR "staff factor" OR "staff factors" OR "motivational factor" OR "motivational factors" OR "motivation factor" OR "motivation factors" OR "motivating factor" OR "motivating factors" OR "motivated factor" OR "motivated factors" OR "motivator factor" OR "motivator factors" OR "external factor" OR "external factors" OR "outer factor" OR "outer factors" OR "internal factor" OR "internal factors" OR "inner factor" OR "inner factors" OR "institutional factor" OR "institutional factors" OR "influence factor" OR "influence factors" OR "factor of influence" OR "factors of influence" OR "induction factor" OR "induction factors" OR "interfering factor" OR "interfering factors" OR "interference factor" OR "interference factors") **OR** MW("associate factor" OR "associates factors" OR "associated factor" OR "associated factors" OR "factor associated" OR "factors associated" OR "related factor" OR "related factors" OR "factor related" OR "factors related" OR "correlated factor" OR "correlated factors" OR "factor correlated" OR "factors correlated" OR "co-related factor" OR "co-related factors" OR "factor co-related" OR "factors co-related" OR "intrinsic factor" OR "intrinsic factors" OR "extrinsic factor" OR "extrinsic factors" OR "individual factor" OR "individual factors" OR "personal factor" OR "personal factors" OR "staff factor" OR "staff factors" OR "motivational factor" OR "motivational factors" OR "motivation factor" OR "motivation factors" OR "motivating factor" OR "motivating factors" OR "motivated factor" OR "motivated factors" OR "motivator factor" OR "motivator factors" OR "external factor" OR "external factors" OR "outer factor" OR "outer factors" OR "internal factor" OR "internal factors" OR "inner factor" OR "inner factors" OR "institutional factor" OR "institutional factors" OR "influence factor" OR "influence factors" OR "factor of influence" OR "factors of influence" OR "induction factor" OR "induction factors" OR "interfering factor" OR "interfering factors" OR "interference factor" OR "interference factors")
#3<br>(Return: ‹*n_3*›)	TI(((scientific OR science OR research) AND (initiation OR education* OR literacy OR training OR abilit* OR vocation* OR skill* OR instruction* OR background OR internship* OR scholarship* OR studentship* OR "talent development")) OR (scientific AND research)) **OR** AB(((scientific OR science OR research) AND (initiation OR education* OR literacy OR training OR abilit* OR vocation* OR skill* OR instruction* OR background OR internship* OR scholarship* OR studentship* OR "talent development")) OR (scientific AND research)) **OR** MW(((scientific OR science OR research) AND (initiation OR education* OR literacy OR training OR abilit* OR vocation* OR skill* OR instruction* OR background OR internship* OR scholarship* OR studentship* OR "talent development")) OR (scientific AND research))
#4<br>(Return: ‹*n_4*›)	TI((education* AND institut*) OR facult* OR universit* OR college* OR "higher education" OR "secondary education" OR "high school" OR "secondary school") **OR** AB((education* AND institut*) OR facult* OR universit* OR college* OR "higher education" OR "secondary education" OR "high school" OR "secondary school") **OR** MW((education* AND institut*) OR facult* OR universit* OR college* OR "higher education" OR "secondary education" OR "high school" OR "secondary school")
#5<br>(Return: ‹*n_5*›)	(#1) AND (#2) AND (#3) AND (#4)

Search refinement:

1. Search Expanders: Apply related words
   and Apply equivalent subjects
2. Types of documents: Academic journals

Return after refinement:

‹*N*› studies in the test carried out in ‹*date*›.

PsycInfo® and PsycArticles® via APA PsycNet® interface	
**Blocks and Returns**	**Search strings**
#1<br>(Return: ‹n_1›)	(Title: (researcher* OR mentor* OR supervisor* OR adviser* OR advisor* OR scientist* OR professor*)) **OR** (Abstract: (researcher* OR mentor* OR supervisor* OR adviser* OR advisor* OR scientist* OR professor*)) **OR** (Keywords: (researcher* OR mentor* OR supervisor* OR adviser* OR advisor* OR scientist* OR professor*))
#2<br>(Return: ‹n_2›)	(Title: ("associate factor" OR "associates factors" OR "associated factor" OR "associated factors" OR "factor associated" OR "factors associated" OR "related factor" OR "related factors" OR "factor related" OR "factors related" OR "correlated factor" OR "correlated factors" OR "factor correlated" OR "factors correlated" OR "co-related factor" OR "co-related factors" OR "factor co-related" OR "factors co-related" OR "intrinsic factor" OR "intrinsic factors" OR "extrinsic factor" OR "extrinsic factors" OR "individual factor" OR "individual factors" OR "personal factor" OR "personal factors" OR "staff factor" OR "staff factors" OR "motivational factor" OR "motivational factors" OR "motivation factor" OR "motivation factors" OR "motivating factor" OR "motivating factors" OR "motivated factor" OR "motivated factors" OR "motivator factor" OR "motivator factors" OR "external factor" OR "external factors" OR "outer factor" OR "outer factors" OR "internal factor" OR "internal factors" OR "inner factor" OR "inner factors" OR "institutional factor" OR "institutional factors" OR "influence factor" OR "influence factors" OR "factor of influence" OR "factors of influence" OR "induction factor" OR "induction factors" OR "interfering factor" OR "interfering factors" OR "interference factor" OR "interference factors")) **OR** (Abstract: ("associate factor" OR "associates factors" OR "associated factor" OR "associated factors" OR "factor associated" OR "factors associated" OR "related factor" OR "related factors" OR "factor related" OR "factors related" OR "correlated factor" OR "correlated factors" OR "factor correlated" OR "factors correlated" OR "co-related factor" OR "co-related factors" OR "factor co-related" OR "factors co-related" OR "intrinsic factor" OR "intrinsic factors" OR "extrinsic factor" OR "extrinsic factors" OR "individual factor" OR "individual factors" OR "personal factor" OR "personal factors" OR "staff factor" OR "staff factors" OR "motivational factor" OR "motivational factors" OR "motivation factor" OR "motivation factors" OR "motivating factor" OR "motivating factors" OR "motivated factor" OR "motivated factors" OR "motivator factor" OR "motivator factors" OR "external factor" OR "external factors" OR "outer factor" OR "outer factors" OR "internal factor" OR "internal factors" OR "inner factor" OR "inner factors" OR "institutional factor" OR "institutional factors" OR "influence factor" OR "influence factors" OR "factor of influence" OR "factors of influence" OR "induction factor" OR "induction factors" OR "interfering factor" OR "interfering factors" OR "interference factor" OR "interference factors")) **OR** (Keywords: ("associate factor" OR "associates factors" OR "associated factor" OR "associated factors" OR "factor associated" OR "factors associated" OR "related factor" OR "related factors" OR "factor related" OR "factors related" OR "correlated factor" OR "correlated factors" OR "factor correlated" OR "factors correlated" OR "co-related factor" OR "co-related factors" OR "factor co-related" OR "factors co-related" OR "intrinsic factor" OR "intrinsic factors" OR "extrinsic factor" OR "extrinsic factors" OR "individual factor" OR "individual factors" OR "personal factor" OR "personal factors" OR "staff factor" OR "staff factors" OR "motivational factor" OR "motivational factors" OR "motivation factor" OR "motivation factors" OR "motivating factor" OR "motivating factors" OR "motivated factor" OR "motivated factors" OR "motivator factor" OR "motivator factors" OR "external factor" OR "external factors" OR "outer factor" OR "outer factors" OR "internal factor" OR "internal factors" OR "inner factor" OR "inner factors" OR "institutional factor" OR "institutional factors" OR "influence factor" OR "influence factors" OR "factor of influence" OR "factors of influence" OR "induction factor" OR "induction factors" OR "interfering factor" OR "interfering factors" OR "interference factor" OR "interference factors"))
#3<br>(Return: ‹n_3›)	(Title: (((scientific OR science OR research) AND (initiation OR education* OR literacy OR training OR abilit* OR vocation* OR skill* OR instruction* OR background OR internship* OR scholarship* OR studentship* OR "talent development")) OR (scientific AND research))) **OR** (Abstract: (((scientific OR science OR research) AND (initiation OR education* OR literacy OR training OR abilit* OR vocation* OR skill* OR instruction* OR background OR internship* OR scholarship* OR studentship* OR "talent development")) OR (scientific AND research))) **OR** (Keywords: (((scientific OR science OR research) AND (initiation OR education* OR literacy OR training OR abilit* OR vocation* OR skill* OR instruction* OR background OR internship* OR scholarship* OR studentship* OR "talent development")) OR (scientific AND research)))
#4<br>(Return: ‹n_4›)	(Title: ((education* AND institut*) OR facult* OR universit* OR college* OR "higher education" OR "secondary education" OR "high school" OR "secondary school")) **OR** (Abstract: ((education* AND institut*) OR facult* OR universit* OR college* OR "higher education" OR "secondary education" OR "high school" OR "secondary school")) **OR** (Keywords: ((education* AND institut*) OR facult* OR universit* OR college* OR "higher education" OR "secondary education" OR "high school" OR "secondary school"))
#5<br>(Return: ‹n_5›)	(#1) AND (#2) AND (#3) AND (#4)
	**Search refinement**:<br>1. Document type: Journal Article<br>2. Publication type: Peer Reviewed Journal<br>**Return after refinement**:<br>‹*N*› studies in the test carried out in ‹*date*›.
